# Nanoparticles revolution: The role of gold and chitosan in endodontics irrigation

**DOI:** 10.6026/973206300211239

**Published:** 2025-05-31

**Authors:** Dipali Dinkar Deshpande, Varsha Pandit, Dhananjay Gunawat, Gauri Sanpurkar

**Affiliations:** 1 Department of Conservative Dentistry and Endodontics, Bharti Vidyapeeth Deemed to be University Dental College and Hospital, Pune, India; 2 Department of Conservative Dentistry and Endodontics, Aditya Dental College, Beed, Maharashtra, India; 3 Department of Oral Medicine and Radiology, Private Practitioner, Chhatrapati, Sambhajinagar, New Delhi, India

**Keywords:** Nanoparticles, endodontics irrigation, anti-inflammatory, biocompatibility

## Abstract

The field of endodontic irrigation has seen nanoparticle innovations which offer improved disinfection together with better clinical treatment results. Nanoparticles composed of gold and chitosan show significant antibacterial properties as well as anti-inflammatory characteristics and biocompatibility potential. The photo thermal properties and catalytic properties are limited to gold nanoparticles whereas the natural antimicrobial capabilities and adhesive properties are limited to chitosan nanoparticles. Nanoparticles play an essential role in endodontic irrigation. However, more clinical work needs to be done to create standardized procedures using these new agents.

## Background:

The goal of endodontic treatment is to eradicate harmful microorganisms from root canal systems completely. The irrigation process is a vital step in the endodontic treatment [1]. Irrigation completes the role of mechanical instrumentation through its ability to eliminate biofilms and dissolve organic tissues and remove debris from the root canal system [2]. The currently utilized sodium hypochlorite (NaOCl), chlorhexidine (CHX) and EDTA irrigating solutions demonstrate multiple drawbacks that include toxicity effects to surrounding tissues and limited penetration along with a reduced capability to combat already developed biofilms [3]. The progress of nanotechnology has elevated the utilization of nanoparticles specifically for endodontic irrigation procedures [4]. Chitosan nanoparticles along with Gold nanoparticles function as promising additions or substitutes for current endodontic irrigating solutions. Nanoparticles exhibit two key properties that allow them to fight microbial biofilms effectively while providing better disinfection in the root canal system: their small scale dimensions and unique physical chemistry attributes and their capability to be biologically friendly [5-6].

## Need for nanoparticles in endodontic irrigation:

New materials demand investigation because traditional irrigants have proved inadequate at effective disinfection. Technical obstacles during root canal irrigation originate from incomplete navigation through complex canal structures combined with poor biofilm destruction particularly in hardened biofilms as well as security concerns linked to chemical substances used in traditional treatments along with their inability to deliver persistent antimicrobial benefit. The potential solution arises from nanotechnology because nanoparticles demonstrate powerful disinfectant capabilities along with deep penetration into dentinal tubules and their large surface area-to-volume ratios [7]. The engineered nanomaterials demonstrate multiple functions that incorporate their delivery of antimicrobial agents while demonstrating independent antimicrobial properties and membrane-interaction effects that destroy cells by lysis [8]. Scientists investigate various nanoparticles but focus especially on gold and chitosan nanoparticles since these demonstrate unique properties. Gold nanoparticles possess antimicrobial properties because they can selectively target bacteria and exhibit both plasmonic behavior and simple modification capabilities which increases their effectiveness [9]. Natural polymer Chitosan shows antimicrobial active properties combined with low toxicity rates and it functions well with living systems. Endodontic irrigation using these substances achieves better antimicrobial effects against the difficult to treat endodontic microorganism *Enterococcus faecalis* [6].

## Overview of gold nanoparticles (AuNPs):

Small sized gold particles known as Gold nanoparticles (AuNPs) exist between 1 to 100 nanometers in dimensions while showcasing exclusive properties in optics and electronics with biological functions [10]. Medical professionals utilize these nanoparticles for different biomedical applications which extend from diagnostics through drug delivery systems and antimicrobial treatment methods [11]. Biocompatible properties together with stability and strong antimicrobial action make AuNPs effective agents for endodontics irrigation particularly when used alone or with additional flushing solutions [9].

## Synthesis and properties:

Synthesis of gold nanoparticles happens through physical, chemical and biological procedures. Scientists commonly perform the reduction of gold salts with sodium citrate or plant extracts as their main chemical synthesis approach [12]. Several properties of AuNPs that emerge during synthesis determine both their disinfectant power and their penetration capacity within dentinal tubules [13]. The strong surface plasmon resonance property of AuNPs makes them highly capable of efficiently absorbing and scattering light. Light activation of AuNPs produces localized heat for photo thermal therapy thus enabling the destruction of bacterial cells through this mechanism [13, 14].

## Antimicrobial mechanism:

The Gold nanoparticles (AuNPs) show antimicrobial activity through various synergizing mechanisms which reinforce their effectiveness at killing bacteria. The main way AuNPs fight bacteria consists of damaging bacterial membranes through direct contact that disrupts cell wall structure with consequent permeability increases leading to cellular destruction [15]. The activity of AuNPs leads to the generation of reactive oxygen species which cause oxidative stress which damages essential components of microbial cells including DNA and proteins. Antibacterial enzyme blockade constitutes another essential mechanism that halts both metabolic processes and DNA replication resulting in bacterial mortality. The capacity of gold nanoparticles exists to penetrate biofilms and destroy resistant microbial structures which normally stay untouched by conventional antimicrobial treatments [16]. The process of breaking down biofilms through AuNPs results in better bacterial response to additional antimicrobial treatments thus providing enhanced infection treatment capabilities. Researchers have proven that AuNPs show effectiveness against *E. faecalis* together with Staphylococcus aureus and several endodontic pathogens. Photo thermal properties enable AuNPs to work more effectively when they are combined with lasers or light sources for root canal disinfection purposes [16].

## Overview of chitosan nanoparticles:

Chitosan derives from the natural polysaccharide compound which scientists extract from the crustacean exoskeletons through chitin. Research in medical fields, along with dental applications, shows enthusiastic interest in using chitosan because of its natural compatibility with human body tissues, its ability to break down in biological systems and its antimicrobial actions [17]. The endodontic irrigation benefit from chitosan nanoparticles (CS-NPs) created through ionic gelation techniques along with emulsion cross-linking because they have microscale dimensions along with high surface contact abilities [6].

## Properties and preparation:

Embodied within the scale of chitosan nanoparticles are dimensions between 20 and 200 nm while each nanoparticle carries a positive electrostatic field. The cationic property of chitosan nanoparticles allows them to tightly bind themselves to negatively charged microbial surfaces together with dentin tissues [18]. Chitosan creates metal ion chelates and shows excellent endotoxin-binding properties thereby contributing to its detoxification capabilities within the root canal system [18]. The small chitosan particles increase solubility while increasing their potential to bind with microbial cells at rates exceeding bulk chitosan tructures. The solvent properties of chitosan improve the effectiveness of both the irrigant solution as well as the delivery of therapeutic agents [19- 20].

## Antimicrobial mechanism:

CS-NPs have multiple antimicrobial mechanisms that result in effective endodontic performance. Membrane disruption occurs as the positive charge present on CS-NP particles enables them to react with negative bacterial membranes thus increasing membrane permeability and releasing harmful contents from inside the cells. The nutrient-chelating property of chitosan removes vital components along with trace minerals that bacteria need to grow and survive [21]. The inhibitory influence of CS-NPs extends to biofilms through their dual ability to impede development and penetrate existing structures to break down the biofilm structure which produces increased bacterial vulnerability. Chitosan demonstrates an additional benefit through its ability to store antimicrobial agents within capsules that allow extended therapeutic effectiveness through controlled drug delivery [22]. The wound-healing along with anti-inflammatory properties displayed by chitosan help support periapical tissues, repair following endodontic procedures to achieve successful outcomes.

## Mechanisms of action:

The effectiveness of gold and chitosan nanoparticles as endodontic irrigants stems from their influence on microorganisms through different specific and collaborative modes of action. Scientific analysis of these mechanisms demonstrates their appropriateness for clinical utilization of gold nanoparticles.

## Gold nanoparticles:

The antimicrobial function of gold nanoparticles operates via following mechanisms [23]. Cell membrane disruption- When present in solutions AuNPs stick to bacterial a wall which subsequently leads to membrane deterioration together with an elevation in membrane permeability. Cellular essential substances leak out as a consequence of this phenomenon. Intracellular penetration- The nano-scale dimension of AuNPs allows these particles to penetrate microbial cells and simultaneously disrupt cellular processes at the vital level. Oxidative strss induction- AuNPs possess the capability to induce oxidative stress by catalyzing the formation of reactive oxygen species (ROS) which results in DNA lipid and protein damage. Photo thermal effect- It generates localized heat from AuNPs when they receive particular light waves for bacteria death specifically in biofilms and distant canal locations.

## Chitosan nanoparticles:

The antimicrobial aspects of chitosan nanoparticles operate through physical interference together with chemical mechanisms to fight bacterial infections [24]. Electrostatic interaction - The positively charged nature of CS-NPs enables strong membrane-binding to negative bacterial membranes thus causing destructive effects on cellular structures. Metal ion chelation - The essential metal ions calcium and magnesium are bound by CS-NPs which lead to disruption of bacterial metabolism. Interference with DNA-DNA transmission becomes impaired through chitosan which enters microbial cells to attach to DNA and stops the bacterial processes of replication and transcription. Biofilm inhibition- CS-NPs show two inhibitory effects on biofilms because they minimize bacterial colonies development and break down substances that protect bacterial communities leading to decreased pathogen resistance. These nanoparticles demonstrate stronger antimicrobial effects when combined with common endodontic irrigants as well as other nanoparticles through a cooperative mechanism [18].

## Comparative efficacy:

Multiple laboratory tests and real-life experiments have researched and contrasted the antimicrobial properties of gold particles together with chitosan nanoparticles against regular endodontic-causing microorganisms.

## Against *Enterococcus faecalis*:

The root canal failure pathogen *E. faecalis* exists as a facultative anaerobic gram-positive bacterium. Studies show antibacterial activity from AuNPs together with CS-NPs reaches high levels when treating this organism [25].

[1] Gold nanoparticles - Low-concentration AuNPs demonstrate substantial capability for reducing *E. faecalis* CFUs coupled with biofilm destruction as an effect of laser irradiation.

[2] Chitosan Nanoparticles - It demonstrate better biofilm disruption abilities together with extended antimicrobial activity because of their adhesive properties and their controlled release pattern.

## Penetration ability:

The success of any irrigation solution depends first and foremost on its capacity to reach and access both dentinal tubules and irregularities within the canal [26]. Small-sized inert AuNPs penetrate dentinal tubules deeply because of their minimal dimensions. While the larger size of CS-NPs provides them with better adherence to dentin walls while extending their antimicrobial retention time.

## Cytotoxicity and biocompatibility:

[1] Gold nanoparticles- The biomedical properties of AuNPs remain acceptable because these nanoparticles demonstrate both minimal toxicity levels in appropriate concentrations along with non-aggressive behaviors within tissues. Prolonged accumulations of these nano-particles remain to be a concern for medical practitioners [27].

[2] Chitosan nanoparticles - The intracanal application benefit from CS-NPs due to chitosan being a naturally derived substance with excellent compatibility and low toxicity at higher doses [28].

## Synergistic use or combinations:

Scientists have studied the antimicrobial effects that come from mixing nanoparticles with other antiseptic agents and treatment mediums.

## Gold nanoparticles with lasers:

AuNPs show great promise as Photo thermal agents for medical use in therapy. Biofilm bacteria and deeply recessed regions in the canal undergo destruction under localized thermal energy from AuNPs which is generated after AuNP exposure to light or laser energy. The combined application of nanoparticles and lasers has proven successful for increasing disinfection effectiveness particularly within tight or bending canal areas [29].

## Chitosan nanoparticles with other agents:

The delivery system of CS-NPs contains multiple possible antimicrobial compounds which include:

[1] Essential oils

[2] Silver nanoparticles

[3] Antibiotics or peptides [30].

When combined with the encapsulated agent chitosan demonstrates a dual antibacterial property which boosts bacterial removal while minimizing antimicrobial resistance risks.

## Sequential or combined use:

A combination of AuNPs followed by CS-NPs or their use as formulation composites offers multiple advantageous outcomes.

[1] Immediate microbial killing by AuNPs.

[2] Prolonged, bioadhesive and anti-inflammatory action by CS-NPs.

Current emerging research about these applications suggests that microbial destruction becomes more effective and tissue connection maintains greater compatibility.

## Advantages and limitations:

## Advantages:

These alternative endodontic treatment agents demonstrate superior features than conventional methods due to their properties as gold and chitosan nanoparticles.

## Gold nanoparticles (AuNPs):

[1] High antimicrobial efficacy, especially against resistant strains like *E. faecalis*.

[2] The Photo thermal properties of nanoparticles allow better microbial destruction when operators apply light exposure.

[3] Excellent penetration into dentinal tubules due to small particle size.

[4] The nanoparticles allow surface modification to bring in therapeutic agents [31].

## Chitosan nanoparticles (CS-NPs) [6]:

[1] These nanoparticles demonstrate properties which make them suitable for periapical tissue applications because they maintain biocompatibility and biodegradability.

[2] Natural origin, reducing potential toxicity.

[3] The bioadhesive properties maintain a lengthy bond between the materials and surrounding dentin and tissues.

[4] Wound healing occurs better after treatment through the combination of anti-inflammatory effects and tissue regeneration abilities.

[5] These substances demonstrate controlled drug release capabilities, which enable their use as drug delivery carriers.

## Limitations:

Some technical and operational barriers exist despite their attractive potential [31].

## Gold nanoparticles:

[1] High cost of synthesis and functionalization

[2] Backed tissues may accumulate these elements if appropriate removal methods are not available.

[3] The investigation of long-term safety risks remains insufficient.

[4] To achieve maximum Photo thermal benefit gold nanoparticles need additional supporting devices such as lasers.

## Chitosan nanoparticles:

[1] The antibacterial strength of this material depends on its molecular weight together with the extent to which it exhibits deacetylation.

[2] Their penetration ability trails behind the penetration rate of AuNPs because of their larger size.

[3] The stability of these particles in solution depends on the pH conditions alongside ionic strength [6].

[4] Limited standardization in preparation methods.

## Current research and clinical implications:

Research based on nanoparticles in endodontics has disclosed optimistic results primarily through in vitro and animal experimentation. Consistent research findings demonstrate that gold nanoparticles (AuNPs) and chitosan nanoparticles (CS-NPs) reduce colony-forming units (CFUs) while also being effective in destroying biofilms. The research study found that chitosan nanoparticles held a comparable effect to sodium hypochlorite standards yet provided better compatibility making them a suitable replacement. Research using AuNPs together with diode lasers demonstrated that they could eliminate most *Enterococcus faecalis* bacteria in tooth extraction models thus demonstrating enhanced treatment potential. New pilot research shows promising treatment results using nanoparticles which cause few adverse side effects Specialists in endodontics promote the inclusion of nanoparticles for endodontic irrigation procedures because of their antimicrobial power and biocompatible nature although clinical protocols have not spread universally [31-32].

## Future prospects:

Nanotechnology integration with biological treatment strategies will shape endodontic irrigation systems through innovative solutions awaiting us in the future. Scientists have started developing combination irrigant systems that unite nanoparticles with standard solutions for better antimicrobial action without compromising the natural compatibility of the materials. Scientists can craft personalized nanomedicine treatments through precise selection of nanoparticle substances based on individual patient microbiota to deliver better targeted medical effects [33]. Scientists are focusing on green synthesis methods which produce nanoparticles through plant-based materials because these methods decrease toxicity while creating less environmental impact. The advancement of nanoparticle therapy requires additional research through in vivo trials and clinical tests to develop standardized procedures as well as safety protocols and extended treatment assessment. The objective now focuses on developing nanoparticles which integrate antimicrobial functions with anti-inflammatory characteristics and remineralization capabilities to treat endodontic conditions by single methodology. The development of delivery systems that include gels as well as sprays and sustained-release formulations will enhance the usability of nanoparticle-based irrigation technology inside clinical practices for better patient results.

## Conclusion:

The characteristics of Gold and Chitosan nanoparticles which include antibacterial potency, tissue compatibility and extended activity characteristics make them suitable choices for modern irrigation methods. Gold and chitosan nanoparticles hold promise as effective complementary agents to conventional irrigation solutions or suitable replacements into the regular dental care procedures.
